# Increased risk of preeclampsia after use of paracetamol during pregnancy – causal or coincidence?

**DOI:** 10.1186/s12884-020-03490-x

**Published:** 2021-01-06

**Authors:** Hetti von Hellens, Leea Keski-Nisula, Heidi Sahlman

**Affiliations:** 1 Institute of Clinical Medicine, School of Medicine, University of Eastern Finland, P.O. Box 1627, FI-70211 Kuopio, Finland; 2 Department of Obstetrics and Gynecology, Kuopio University Hospital, Puijonlaaksontie 2, 70210 Kuopio, Finland; 3 School of Pharmacy, University of Eastern Finland, P.O. Box 1627, FI-70211 Kuopio, Finland

**Keywords:** Acetaminophen, Paracetamol, Preeclampsia, Pregnancy

## Abstract

**Background:**

The maternal use of paracetamol during pregnancy has been associated with the development of preeclampsia. This study aims to clarify whether the connection is causal or whether it is due to reverse causation.

**Methods:**

This study is a continuation of the retrospective case cohort study examining 2,508 pregnant women using a variety of drugs and the development of preeclampsia (1,252 women with preeclampsia and 1,256 controls). For the purposes of this study, more precise data was collected from several hospital databases of the women among this cohort who had reported taking paracetamol during pregnancy (indications, gestational period etc.); this was evaluated in association with the development of preeclampsia.

**Results:**

5.5% (100 cases and 37 controls) of all the study population (2,508) had clearly reported paracetamol use. Women with preeclampsia had used significantly more often paracetamol during pregnancy compared to controls (cases 8.0%, controls 2.9%, p < 0.001). The difference was most evident in the third trimester (after the 29th GW) and the use of paracetamol was associated with both mild and severe preeclampsia. Headache and “general pain” were the most common indications for medication among all paracetamol users.

**Conclusions:**

The use of paracetamol in the third trimester of pregnancy was associated with preeclampsia. This observation indicates that association between paracetamol use and preeclampsia is probably due to reverse causation, i.e. women with preeclampsia experience more headaches due to preeclampsia symptoms since this association was not detected with the use of paracetamol in earlier stages of pregnancy.

## Background

Paracetamol, also known as acetaminophen, is an analgesic commonly used to treat pain and fever. In general, it is considered to be quite safe without significant side-effects and thus it is also widely used during pregnancy [1]. Though it possesses analgesic and antipyretic properties, the precise mechanism of action of paracetamol is not fully understood [2]. It has a complex analgesic mechanism acting on various levels of pain stimulus conduction such as tissue receptors, spinal cord, thalamus and cerebral cortex. Similarly to non-steroidal anti-inflammatory drugs (NSAIDs), paracetamol suppresses prostaglandin production but does not evoke the typical gastrointestinal side effects associated with NSAIDs [3].

In recent studies, maternal paracetamol use during pregnancy has been linked with possible long term side-effects on the developing fetus and later in childhood, such as the development of asthma, lower IQ, autism spectrum disorder, attention-deficit/hyperactivity disorder, poorer attention and executive functions as well as behavioral problems [4–7]. In addition to the possible side-effects on the fetus, the use of paracetamol has been linked to a risk of preeclampsia [8–10].

Preeclampsia is a rather common heterogeneous disease, occurring in 3 − 8% in pregnancies worldwide [11] which can develop into a lethal multiorgan syndrome [12]. The clinical features of the disease involve newly-onset hypertension and proteinuria but the specific definition for preeclampsia varies according to different guidelines and between countries [13]. The pathogenesis of preeclampsia is still unclear, and the main approach to prevent maternal morbidity is the clinical management of its symptoms [12]. Nevertheless, it is known that preeclampsia is, at least partly, related to abnormal placentation and further placental dysfunction, and the fetus is potentially susceptible to the effects of uteroplacental disturbances, such as fetal growth restriction or placental abruption [13]. Preeclampsia is also predictive of a higher risk for cardiovascular and metabolic disease later in a woman’s life [12, 14, 15].

It is worth noting that the duration and the time of the gestational drug exposure may have a significant effect on the fetal risk [5]. However, the relevance of these studies in relation to clinical practice is still questionable and more research is required. There is also an extensive literature indicating that paracetamol is a safe drug for both the mother and fetus when used prenatally to treat mild-to-moderate pain [16]. Moreover, in Finland, paracetamol is the recommended medication for relieving mild pain and fever during pregnancy [17]. Black and associates [16] concluded that further research into the safety of different analgesics used during pregnancy, including paracetamol, is still urgently required since questions have been raised about their safety with regards to long-term fetal effects.

It has been shown by Sahlman and associates [10] that women with preeclampsia used various kinds of drugs significantly more often during the index pregnancy compared to women without preeclampsia and that paracetamol was one of those drugs. The women experiencing preeclampsia had used paracetamol prenatally nearly 2.5 times more often than the controls [10]. This raised the question if there was a causal connection between paracetamol use and the occurrence of preeclampsia, or was the association merely attributable to reverse causation, i.e. women with preeclampsia suffer more headaches and other symptoms of preeclampsia and consequently take more pain-relieving medication. Thus, we wanted to examine in more detail the indications for paracetamol use in our birth register cohort data and to evaluate more closely this apparent association between the use of paracetamol and the development of preeclampsia.

## Methods

This study was a retrospective cohort study composed originally from data provided by the Kuopio University Hospital (KUH) birth register, which houses information about all the women who gave birth in Kuopio University Hospital in years between 2002 and 2016. KUH is a tertiary hospital where the annual delivery rate on average was approximately 2500 births during the study years. We used the same cohort as Sahlman and associates [10] but examined more closely the hospital records of those women reported having taken paracetamol during their pregnancy. One author (HvH) examined medical records of the each individual woman who had had preeclampsia and had reported paracetamol use during pregnancy (cases), and also of the women in a control group, i.e. women who had used paracetamol but did not suffer from preeclampsia (controls). Women were selected from the birth register in accordance to their International Statistical Classification of Diseases and Related Health Problems 10th revision (ICD-10) diagnosis during pregnancy and delivery (see [10]).

**Fig. 1:**
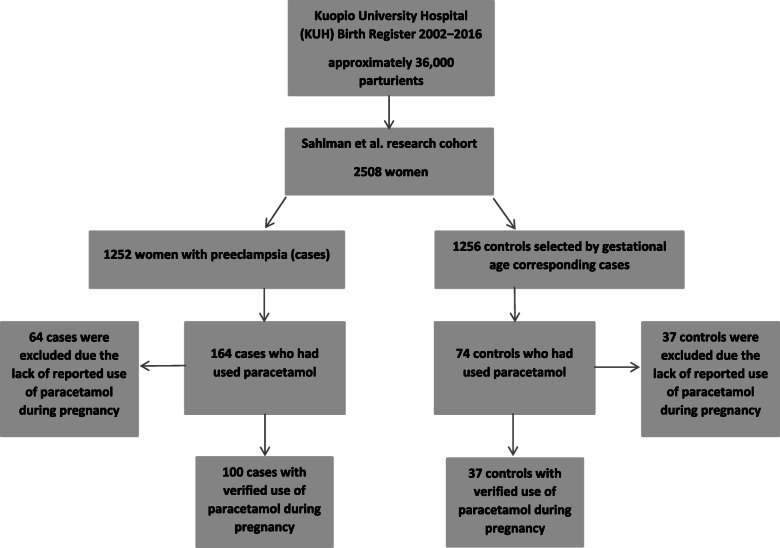
Flowchart demonstrating exclusions and group division of the study cohort

Information about the drug use during pregnancy was collected from KUH birth registers in which the information of the use of any medication during pregnancy is self-reported during the ongoing pregnancy but which also holds the possibility to include medications taken during the perinatal period in hospital. In the earlier study [10] attempts were made to exclude those women with peripartum drug use on the basis of register data; thus the association between the use of paracetamol during pregnancy and the development of preeclampsia was clarified. Here, we re-examined the use of paracetamol based on each individual patient’s hospital records, especially the information regarding the timing and the possible indications of paracetamol use were also recorded. The flow chart of the study participants and the subdivision of the groups are shown in Fig. 1.

In addition, we determined from the hospital records whether the self-reported data of the use of paracetamol had occurred soon before birth, during parturition or even after birth. Women who had reported the use of paracetamol solely during and after birth were excluded from the paracetamol users evaluated in this study. Furthermore, other information was also collected including the dose of paracetamol, whether its use was regular or irregular and the date when the preeclampsia diagnosis was given or the suspicion of preeclampsia had first been noted. This data was collected by obtaining information from each woman’s individual medical records concerning the pregnancy (including also birth registers). In these rechecks, we noticed that several of those women who had reported the use of paracetamol during pregnancy had only recorded use during or after the birth. As stated, these individuals were excluded from the paracetamol users within the context of this study, because we were interested in the earlier use of paracetamol during pregnancy. Thus, from the original group of paracetamol users during pregnancy (*N* = 238), 101 women were excluded (preeclampsia group *N* = 64, control group *N* = 37) (Fig. 1).

The preceding study by Sahlman and associates [10] has been approved by Central Finland Health Care District ethical committee (18U/2011, 6.10.2016) and that clearance applied to this continuation study since the same cohort was used and no new participants were included.

### Statistical analysis

Descriptive statistics or crosstabs analyses were utilized to assess values and percentages of cases and controls. P-values were calculated by Pearson Chi-square test or Fisher’s exact test. The relationships between preeclampsia and the use of paracetamol were analyzed by binary logistic regression or multinomial logistic regression to compute the odds ratio (OR)/adjusted odds ratio (aOR) and confidence intervals 95% (CI 95%). Data was adjusted with maternal age, parity, BMI, smoking, diagnosed hypertension and diabetes or gestational diabetes mellitus (GDM) (see details [10]). A *p*-value < 0.05 was considered statistically significant. Statistical analysis was performed with the SPSS (version 25) program.

## Results

A total of 238 women out of 2,508 (9.5%) had reported using paracetamol during pregnancy in the self-reported register database. After a strict recheck of the data and the aforementioned exclusions, 137 women (5.5%) (100 preeclampsia cases and 37 controls) had clearly reported the use of paracetamol during pregnancy before the onset of delivery as well as providing information about the indications and the timing of paracetamol use. Even after this recheck, women who developed preeclampsia had used significantly more often paracetamol during pregnancy compared to the controls (cases 8.0%, controls 2.9%, *p* < 0.001).

Among all paracetamol users (*N* = 137), headache and “general pain” were clearly the most common indications for medication (Table 1). Women with preeclampsia suffered more from headaches which they treated with paracetamol as compared to controls (cases 67.0% vs. controls 35.1%, *p* < 0.001). Other indications, including migraine as well as “fever, flu or other” and unknown, were more common in the group of controls than in women with preeclampsia. In general, the majority of women reporting the use of paracetamol had only one recorded indication for paracetamol use. However, women developing preeclampsia more often had more than one indication than the controls (cases 18.0% vs. controls 5.4%).

**Table 1:** Indications for paracetamol use

Indication	n (%)	Pre-eclampsia n (%)	Controls n (%)	*p*-value
**Headache**	80 (58.4)	67 (67.0)	13 (35.1)	0.001^a^
**Pain**	52 (38.0)	35 (35.0)	17 (45.9)	0.241^a^
**Other indications**	25 (18.2)	16 (16.0)	9 (24.3)	0.263^a^
** Migraine**	11 (8.0)	8 (8.0)	3 (8.1)	
** Fever, flu or other**	9 (6.6)	6 (6.0)	3 (8.1)	
** Unknown**	5 (3.7)	2 (2.0)	3 (8.1)	
**Only one indication**	112 (81.8)	80 (80.0)	32 (86.5)	0.052^b^
**More than one indication**	20 (14.6)	18 (18.0)	2 (5.4)	
**Total numbers of women**	137 (100)	100 (100)	37 (100)	

*P*-values are estimated by ^a^Pearson Chi-square test or ^b^Fisher’s exact test

Among all paracetamol users, 32 women (23.4% of the users and 1.3% of all study population) had used paracetamol before the 15th gestational week (GW), 38 women (27.7% of the users and 1.5% of all study population) between 15th -29th GWs and 51 women (37.2% of the users, 2.0% of all study population) after 29th GWs (Tables 2 and 3). Women with preeclampsia had used more paracetamol during all three different gestational periods as compared to the controls, but the difference was most evident and significant in the third trimester (after 29th GWs) (Table 3). In the third trimester, the use of paracetamol was significantly associated with both mild and severe preeclampsia. In an unadjusted analysis, a significant association was also seen between the paracetamol use in 15th -29th GWs and in severe cases of preeclampsia.

**Table 2:** Paracetamol users divided according to trimesters.

**Timing of paracetamol use**	**All n (%)**	**Pre-eclampsia** **n (%)**	**Controls** **n (%)**	***p*-value**^**a)**^
**< 15 GWs**	32(23.4)	19 (19.0)	13 (35.1)	0.047
**≥ 15 and < 29 GWs**	38 (27.7)	25(25.0)	13 (35.1)	0.239
**≥ 29 GWs**	51 (37.2)	45 (45.0)	6 (16.2)	0.002
**Unknown**	16 (11.7)	11 (11.0)	5 (13.5)	0.684
**Total**	137(100.0)	100 (100.0)	37 (100.0)	

^a)^*P*-values are estimated by Pearson Chi-square test, *GWs=* gestational weeks

**Table 3:** Use of paracetamol and the severity of preeclampsia in cases (*n*=1,252) and controls (*n*=1,256).

**Time of use of paracetamol**	**All n (%)**	**Pre-eclampsia n (%)**	**Controls n (%)**	**Unadjusted OR (95% CL)**	***p*-value**	**Adjusted OR (95% CL)**^**a**^	***p*-value**
**< 15 GWs**	32 (1.3)	19 (1.5)	13 (1.0)	1.48 (0.72–3.01)	0.279	1.48 (0.67–3.24)	0.331
		9 (1.2) mild		1.20 (0.51–2.81)	0.680	1.16 (0.46–2.92)	0.752
		10 (1.9) severe		1.88 (0.82–4.32)	0.136	1.95 (0.79–4.80)	0.146
**≥ 15 and < 29 GWs**	38 (1.5)	25 (2.0)	13 (1.0)	1.96 (1.00–3.85)	0.051	2.06 (0.95–4.45)	0.067
		13 (1.8) mild		1.74 (0.80–3.77)	0.161	1.89 (0.81–4.45)	0.143
		12 (2.3) severe		2.27 (1.03–5.00)	0.043	2.30 (0.94–5.60)	0.067
**≥ 29 GWs**	51 (2.0)	45 (3.6)	6 (0.5)	7.81 (3.32–18.37)	< 0.001	7.63 (3.14–18.55)	< 0.001
		29 (4.0) mild		8.65 (3.57–20.93)	< 0.001	8.50 (3.40–21.17)	< 0.001
		16 (3.1) severe		6.64 (2.58–17.07)	< 0.001	6.36 (2.38–16.94)	< 0.001
**Unknown**	16 (0.6)	11 (0.9)	5 (0.4)				
**All paracetamol users**	137(5.5)	100 (8.0)	37 (2.9)	2.86 (1.95–4.21)	< 0.001	2.84 (1.86–4.32)	< 0.001
**All women**	2508 (100)	1252 (100)	1256 (100)				

*CL* confidence limits. *GWs* gestational weeks ^a)^ adjusted for maternal age, parity, BMI, smoking, diagnosed hypertension and diabetes or GDM. *P*-values are estimated by Logistic regression analysis.

Of all paracetamol users, 90.5% had used the drug on an as-needed basis. The doses of the paracetamol used were seldom reported [36.5% of users did not report what kind of dose (mg/day) they took, 54.7% of the users did not state how many doses/day] and the duration of the paracetamol use was usually also not recorded.

## Discussion

In our earlier study, we observed an association between the maternal use of paracetamol and preeclampsia [10]. Our objective in this study was to examine more closely the use of paracetamol during pregnancy and to clarify how probable it would be that there was a causal relationship between these two phenomena. For this purpose, the data concerning the timing of paracetamol use was collected from each individual patient’s hospital medical records. This is the first study which has investigated paracetamol use in all three trimesters and also evaluated the association between the timing of paracetamol use and the severity of preeclampsia.

The results of this study revealed that the women with preeclampsia were using paracetamol more than the controls and this difference was most obvious during the third trimester. A significant association between the use of paracetamol and preeclampsia was seen; this was evident on or after the 29th GW but not in earlier weeks. The results are in line with the earlier study by Rebordosa and associates [18] who reported that paracetamol use at any time during pregnancy or during the third trimester was associated with an increased risk of hypertension or preeclampsia. Scialli and associates [19] also detected an association between an increased risk of preeclampsia and the use of paracetamol in the third trimester. Additionally, the use of paracetamol during the third trimester of pregnancy has been linked to an increased risk of preterm birth following preeclampsia [18].

The pathogenesis of preeclampsia is, at least partly, connected to abnormal placentation and placental dysfunction and thus the first trimester of pregnancy is considered to be the most important time for the development of preeclampsia [11]. Since the development of preeclampsia starts during the early weeks of pregnancy but the first clinical signs of preeclampsia are usually detected only after the 20th GW [20], the use of paracetamol during the early gestational periods is assumed to be the most crucial period of time, if one considers the drug’s role in the development of preeclampsia. Since the association between paracetamol and preeclampsia was most obvious on or after 29th GWs, it is most likely to be due to reverse causation. Headache, is one of the most common symptoms of preeclampsia; it may precede as well accompany preeclampsia [21]. In this study, headache was also the most common indication in both groups however, being more common in the women with preeclampsia.

As well as the timing and the indication of paracetamol use, we also collected data about the dose of paracetamol and the duration of the use. Taking into account the fact that paracetamol is an over-the-counter drug and is usually used only irregularly when needed, it was difficult to determine precise doses and durations of use from the database or in the medical records of the women in the study. Nevertheless, its use was mostly irregular and the dose was usually 500 mg or 1000 mg at one time. Often the exact day when paracetamol had been taken was reported but unfortunately how long the use had continued remained unknown.

It is also important to note that there were probably some women who had used paracetamol during pregnancy but failed to record it into the database. The number of women who reported taking paracetamol was surprisingly low and this was a significant weakness of our study, leading to small subgroups with many unpowered statistical test results. Firstly, this is possibly due to the nature of an over-the-counter, easily available, drug that has been reported to be safe, and secondly, due to the weakness of a retrospective register study. One possible method to gain more precise information about the use of drugs would be a diary-type prospective cohort, in which women could record on a daily basis exact information about their use, dose and length of the use of paracetamol and other drugs. The recent study by Bandoli and associates [22] utilized daily exposure diaries; they reported that as many as 62% of pregnant women had used paracetamol during their pregnancy. Of the women who had reported using paracetamol, over two-thirds reported its use in the first and second trimesters and slightly over every second woman in the third trimester.

One important aspect of this study was that we were able to access each individual patient’s hospital medical records and as such, evaluate more closely the indications and the timing of paracetamol use and subsequently analyze the paracetamol use according to the time of use and the severity of disease. The KUH Birth Register contains information on self-reported use of drugs during pregnancy, which covers also the use of over-the-counter drugs. However, whenever drug use is self-reported by mothers, there is a risk of both recall bias and information bias.

## Conclusions

This retrospective birth register study revealed that there was indeed a significant association between the paracetamol use among pregnant women on and after the 29th GW and the development of preeclampsia. Nonetheless, we propose that this risk was merely attributable to reverse association, although it increased gradually from the early GWs towards the end of pregnancy, but the numbers of those exposed in the early stages of pregnancy were too low to permit drawing any definite conclusions. In the future, more prospective diary exposure studies will be needed to clarify the causal role of early drug exposure and pregnancy complications such as preeclampsia.

## Data Availability

The datasets generated and/or analysed during the current study are not publicly available due to the risk of identifying patients but are available from the corresponding author on reasonable request.

## Abbreviations

aOR: Adjusted odds ratio
CI 95%: Confidence intervals 95%
GDM: Gestational diabetes mellitus
GW: Gestational week
ICD-10: International Statistical Classification of Diseases and Related Health Problems 10th revision
KUH: Kuopio University Hospital
NSAIDs: Anti-inflammatory drugs
OR: Odds ratio

## References

[1] Allegaert K, Mian P, Lapillonne A, van den Anker JN (2019). Maternal paracetamol intake and fetal ductus arteriosus constriction or closure: a case series analysis. Br J Clin Pharmacol.

[2] Anderson BJ (2008). Paracetamol (Acetaminophen): mechanisms of action. Paediatr Anaesth.

[3] Jozwiak-Bebenista M, Nowak JZ (2014). Paracetamol: mechanism of action, applications and safety concern. Acta Pol Pharm.

[4] Toda K (2017). Is acetaminophen safe in pregnancy?. Scand J Pain.

[5] Gou X, Wang Y, Tang Y, Qu Y, Tang J, Shi J, Xiao D, Mu D (2019). Association of maternal prenatal acetaminophen use with the risk of attention deficit/hyperactivity disorder in offspring: A meta-analysis. Aust N Z J Psychiatry.

[6] Chen MH, Pan TL, Wang PW, Hsu JW, Huang KL, Su TP, Li CT, Lin WC, Tsai SJ, Chen TJ, Bai YM. (2019) Prenatal Exposure to Acetaminophen and the Risk of Attention-Deficit/Hyperactivity Disorder: A Nationwide Study in Taiwan. J Clin Psychiatry 80:10.4088/JCP.18m12612. 10.4088/JCP.18m12612 31509360

[7] Ji Y, Azuine RE, Zhang Y, Hou W, Hong X, Wang G, Riley A, Pearson C, Zuckerman B, Wang X (2019). Association of Cord Plasma Biomarkers of In Utero Acetaminophen Exposure With Risk of Attention-Deficit/Hyperactivity Disorder and Autism Spectrum Disorder in Childhood. JAMA Psychiatry.

[8] Blue NR, Murray-Krezan C, Drake-Lavelle S, Weinberg D, Holbrook BD, Katukuri VR, Leeman L, Mozurkewich EL. (2018) Effect of ibuprofen vs acetaminophen on postpartum hypertension in preeclampsia with severe features: a double-masked, randomized controlled trial. Am J Obstet Gynecol 218:616.e1-616.e8. 10.1016/j.ajog.2018.02.016 PMC9097787 29505772

[9] Rebordosa C, Zelop CM, Kogevinas M, Sorensen HT, Olsen J (2010). Use of acetaminophen during pregnancy and risk of preeclampsia, hypertensive and vascular disorders: a birth cohort study. J Matern Fetal Neonatal Med.

[10] Sahlman H, Koponen M, El-Nezami H, Vahakangas K, Keski-Nisula L (2019). Maternal use of drugs and preeclampsia. Br J Clin Pharmacol.

[11] Hod T, Cerdeira AS, Karumanchi SA. (2015) Molecular Mechanisms of Preeclampsia. Cold Spring Harb Perspect Med 5:10.1101/cshperspect.a023473. 10.1101/cshperspect.a023473 PMC4588136 26292986

[12] Steegers EA, von Dadelszen P, Duvekot JJ, Pijnenborg R (2010). Pre-eclampsia. Lancet.

[13] Brown MA, Magee LA, Kenny LC, Karumanchi SA, McCarthy FP, Saito S, Hall DR, Warren CE, Adoyi G, Ishaku S, International Society for the Study of Hypertension in Pregnancy (ISSHP) (2018). Hypertensive Disorders of Pregnancy: ISSHP Classification, Diagnosis, and Management Recommendations for International Practice. Hypertension.

[14] Kajantie E, Eriksson JG, Osmond C, Thornburg K, Barker DJ (2009). Pre-eclampsia is associated with increased risk of stroke in the adult offspring: the Helsinki birth cohort study. Stroke.

[15] Kajantie E, Osmond C, Eriksson JG (2017). Gestational hypertension is associated with increased risk of type 2 diabetes in adult offspring: the Helsinki Birth Cohort Study. Am J Obstet Gynecol.

[16] Black E, Khor KE, Kennedy D, Chutatape A, Sharma S, Vancaillie T, Demirkol A (2019). Medication Use and Pain Management in Pregnancy: A Critical Review. Pain Pract.

[17] Duodecim T. Duodecim medication database. In: Accessed November/30 2019.

[18] Rebordosa C, Kogevinas M, Bech BH, Sorensen HT, Olsen J (2009). Use of acetaminophen during pregnancy and risk of adverse pregnancy outcomes. Int J Epidemiol.

[19] Scialli AR, Ang R, Breitmeyer J, Royal MA (2010). A review of the literature on the effects of acetaminophen on pregnancy outcome. Reprod Toxicol.

[20] Chaiworapongsa T, Chaemsaithong P, Yeo L, Romero R (2014). Pre-eclampsia part 1: current understanding of its pathophysiology. Nat Rev Nephrol.

[21] Chaiworapongsa T, Chaemsaithong P, Korzeniewski SJ, Yeo L, Romero R (2014). Pre-eclampsia part 2: prediction, prevention and management. Nat Rev Nephrol.

[22] Bandoli G, Palmsten K, Chambers C. (2019) Acetaminophen use in pregnancy: Examining prevalence, timing, and indication of use in a prospective birth cohort. Paediatr Perinat Epidemiol. 10.1111/ppe.12595 PMC7192766 31696962

